# The role of familial factors and neuroticism in the association between exposure to offensive behaviors at work and long-term sickness absence due to common mental disorders - a prospective twin study

**DOI:** 10.1186/s12889-024-19000-z

**Published:** 2024-06-01

**Authors:** Maria Wijkander, Pia Svedberg, Jurgita Narusyte, Iman Alaie, Petra Lindfors, Tianwei Xu, Linda L. Magnusson Hanson

**Affiliations:** 1 Stress Research Institute, Department of Psychology, Stockholm University, Stockholm, Sweden; 2 Division of Insurance Medicine, Department of Clinical Neuroscience, Karolinska Institutet, Stockholm, Sweden; 3 Department of Medical Sciences, Child and Adolescent Psychiatry, Uppsala University, Uppsala, Sweden; 4 Department of Psychology, Stockholm University, Stockholm, Sweden

**Keywords:** Mental disorders, Neuroticism, Personality, Sickness absence, Sick leave, Twin study, Work-related bullying, Work-related harassment, Work-related violence

## Abstract

**Objectives:**

The aim of this study was to investigate associations between exposure to work-related violence/threats and harassment, and future sickness absence (SA) due to common mental disorders (CMDs), taking familial factors (shared genetics and early-life environment) and neuroticism into account.

**Methods:**

The study sample included 8795 twin individuals from the Swedish Twin Project of Disability Pension and Sickness Absence (STODS), including survey data from the Study of Twin Adults: Genes and Environment (STAGE). Self-reported work-related violence and/or threats as well as work-related harassment (including bullying) and national register data on SA due to CMDs were analyzed using standard logistic regression, and conditional logistic regression among complete twin pairs discordant on exposures. Individuals were followed for a maximum of 13 years. Interactions between neuroticism and exposures were assessed using both multiplicative and additive interaction analyses.

**Results:**

Exposure to work-related violence/threats was associated with higher odds of SA due to CMDs when adjusting for age, sex, marital status, children, education, type of living area, work characteristics, and symptoms of depression and burnout (OR 2.11, 95% CI 1.52–2.95). Higher odds of SA due to CMDs were also found for exposure to harassment (OR 1.52, 95% CI 1.10–2.11) and a combined indicator of exposure to violence/threats and/or harassment (OR 1.98, 95% CI 1.52–2.59), compared with the unexposed. Analyses of twins discordant on exposure, using the unexposed co-twin as reference, showed reduced ORs. These ORs were still elevated but no longer statistically significant, potentially due to a lack of statistical power. No multiplicative interaction was found between neuroticism and exposure to work-related violence/threats, or harassment. However, a statistically significant additive interaction was found between neuroticism and exposure to violence/threats, indicating higher odds of SA due to CMDs in the group scoring lower on neuroticism.

**Conclusions:**

Exposure to work-related offensive behaviors was associated with SA due to CMDs. However, the results indicated that these associations may be partly confounded by familial factors. In addition, an interaction between exposure and neuroticism was suggested. Thus, when possible, future studies investigating associations and causality between offensive behaviors at work and mental health-related outcomes, should consider familial factors and neuroticism.

**Supplementary Information:**

The online version contains supplementary material available at 10.1186/s12889-024-19000-z.

## Background

Sickness absence is associated with extensive negative consequences, both for the affected individual as well as for society, in terms of production loss and high insurance costs [1]. Previous studies have shown that, from the individual perspective, long, often defined as > 14 days, and recurrent episodes of sickness absence spells are associated with an increased risk of e.g. future unemployment [2], permanent exclusion from the labor market due to disability pension [3–6] and mortality [7–11].

The sickness absence rate is high in Sweden as well as in most other European countries [12, 13]. Common mental disorders (CMDs) are the most common reason for sickness absence in Sweden [14]. CMDs include mood/affective disorders as well as neurotic, stress-related and somatoform disorders. The high proportion of individuals suffering from CMDs impose a great economic burden to European countries, largely due to the indirect costs in terms of social security benefits [13, 15]. Exposure to certain psychosocial work characteristics, such as high job demands and low job control, have been identified as potential risk factors for negative health consequences, including depression and burnout [16] as well as short and long spells of sickness absence, due to mental disorders [17]. Recent studies also suggest that psychosocial working conditions, such as high quantitative demands, low decision authority and job insecurity, are associated with sickness absence of different lengths [18, 19].

Also, exposure to offensive behaviors at work, including work-related violence, threats or harassment and bullying may be important. Previous studies have indicated an association between exposure to work-related offensive behaviors, such as harassment, bullying, violence and threats of different kinds and common mental health problems [20–24]. Moreover, a recent study suggested that exposure to work-related violence and/or threats of violence predicts future long-term sickness absence due to mental disorders [25]. However, challenges remain before concluding the association between exposure to offensive behaviors at work and sickness absence to be causal.

One challenge is related to unknown alternative predictors. For example, the associations between exposure to offensive or abusive behaviors and health outcomes may at least partially be explained by individual characteristics including predispositions [26, 27]. Earlier studies also suggest that exposure to workplace violence or bullying are linked to individual factors such as personality [28], which are largely determined by genetics [29]. Personality is assumed to determine how an individual appraise and act in different situations, including the coping strategies used [30], and can influence the risk of experiencing negative life events [30, 31]. Neuroticism has been consistently associated with distress and negative health outcomes, such as major depressive disorder [30, 32, 33]. In general, individuals high on neuroticism have a tendency to respond to different types of threats, frustration and loss with strong negative emotions and may respond with intense affect in situations which may not evoke such a reaction in others [34], which in turn may contribute to negative mental health outcomes. Neuroticism has also been associated with an increased risk of exposure to negative social behaviors at work [35, 36] and to an increased risk of sickness absence, although the causal relationship remains to be determined [37]. Furthermore, personality can be a potential moderator in associations between work-related social stressors and negative outcomes. One study has e.g. indicated that neuroticism may moderate the relationship between workplace bullying and workplace deviance, such as violation of social norms, and thus threaten employee well-being [38]. But so far studies on the interaction between neuroticism and exposure to work-related stressors and psychological or mental health outcomes are rare.

Moreover, earlier research shows that being exposed to offensive behaviors at work are linked to childhood adversity, including being bullied as a child [39, 40]. A few studies investigating the role of familial factors (genetics and shared environment (primarily family environment while growing up)) in the associations between adverse work characteristics and symptoms of mental disorders as well as sickness absence due to mental disorders suggest that familial factors may have an impact on the associations [41, 42]. However, these studies have focused on low workplace social support. Thus, the role of familial factors in the associations between work-related offensive/abusive behaviors and sickness absence due to CMDs are still to be elucidated.

Genetic factors and early adversity may both be associated with predispositions/vulnerability for mental disorders [40, 43–45], as well as other health outcomes which could be determined by the combination of individual vulnerability and exposure to later stressors such as poor psychosocial working conditions. This means that familial factors may be both confounding factors in the association between psychosocial work characteristics, including exposure to violence and harassment, and sickness absence due to CMDs, or effect modifiers/moderators [46].

In the present prospective twin study, our aim was to investigate if associations between exposure to work-related violence/threats and/or harassment and long-term sickness absence due to CMDs were influenced by familial factors (genetics and shared early environment) and if neuroticism could have an impact on the associations.

This twin study, which was performed with a co-twin control design, sometimes referred to as discordant twin pair design, takes advantage of the fact that monozygotic twin pairs in general share 100% of their genes, while dizygotic twins share on average 50% of the genes, and both types of twins share the same early environment when reared together [47]. Consequently, the unexposed twin serves as a nearly perfect control to the exposed twin [48]. The fact that the twins are matched both on shared environmental and genetic backgrounds is a major strength and makes it possible to control for these potential confounding factors that otherwise may not be possible.

## Materials and methods

### Participants and data sources

The study sample was derived from the Swedish Twin project of Disability pension and Sickness absence (STODS) including both survey- and national registry data. Twins in STODS were identified in the Swedish Twin Registry [49]. In the present study, a sub-cohort from STODS was included in the analyses, comprising twins born between 1959 and 1985 who initially took part in the Study of Swedish Twin Adults: Genes and Environment (STAGE) in 2005–2006 [50]. In the STAGE study > 40 000 Swedish adult twins were invited to participate in a comprehensive survey including measures of environmental exposures, social situation, health, behavior, and individual characteristics such as personality [50]. Data on demographics were obtained from the Longitudinal Integrated Database for Health Insurance and Labor Market Studies (LISA), held by Statistics Sweden containing data collected since year 1990, on all individuals aged ≥ 15 years residing in Sweden [51]. Date of deaths were derived from the Causes of Death registry held by the National Board of Health and Welfare, and consecutive annual data on sickness absence spells, including diagnoses, start and ending dates, were retrieved from the Micro Data for Analysis of Social Insurance (MiDAS) register, held by the Swedish Social Insurance Agency. MIDAS contain all sickness absence spells, that are longer than 14 days for regular employees, and payments of disability pension in Sweden since the year 1994, including information on diagnostic codes on disability pension for all years and sick leave since 2005.

**Fig. 1:**
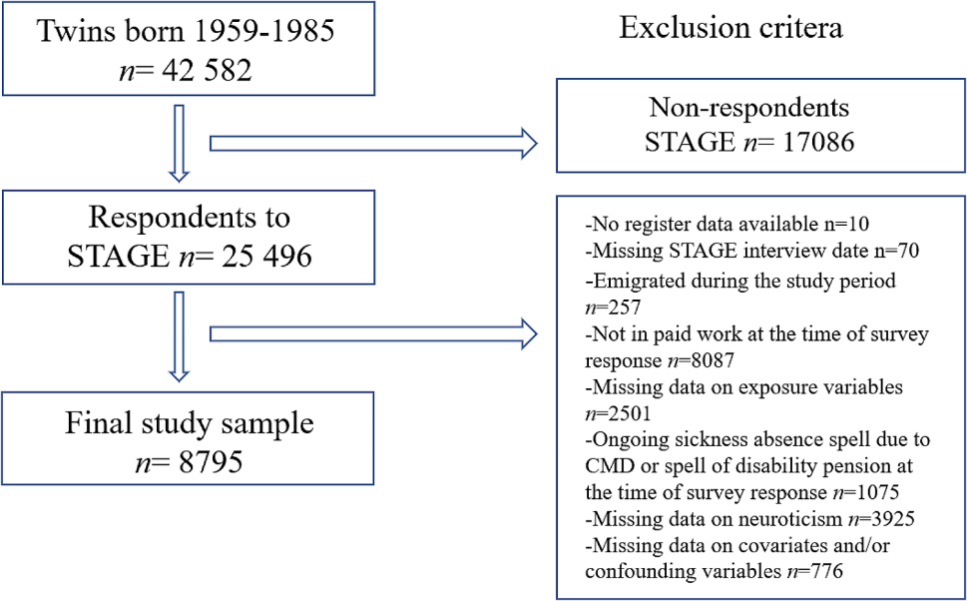
Study population, inclusion and exclusion criteria and the final study sample. STAGE = Study of Twin Adults Genes and Environment

This study included twins who had participated in the STAGE survey and were in paid work (working either full-time or part-time employed in a in a temporary or permanent position, or self-employed) during the time of the survey baseline and the previous 3 years. Exclusion criteria included disability pension or ongoing sickness absence at the time of participation in the STAGE study in 2005–2006, as well as missing information regarding exposures and covariates. The final study sample included *n* = 8795 twin individuals born in Sweden (see Fig. 1 for sample selection and exclusion criteria). In total, there were 2607 complete same-sex twin pairs in the sample. Of these 1516 pairs were monozygotic (MZ), and 1033 dizygotic (DZ). See Table 1 for information regarding zygosity for all twin individuals in the sample. Zygosity was determined by questions regarding the similarity between twins in a pair, a method which has been validated through DNA-analysis and shown to be about 99% accurate [49].

**Table 1:** Characteristics of the whole study sample, stratified on outcome and exposure to work-related offensive behaviors

		Total	Exposure to work-related violence/threats and/or harassment/bullying			No exposure to work-related violence/threats and/or harassment/bullying		
Background factors			All *n* (%)	Sickness absence due to CMD *n* (%)	No sickness absence due to CMD *n* (%)	All *n* (%)	Sickness absence due to CMD *n* (%)	No sickness absence due to CMD *n* (%)
		**8795**	304	121	183	8491	1368	7123
Sex								
	Women	4674 [53]	208 [68]	93 [77]	115 [63]	4466 [53]	993 [73]	3473 [49]
	Men	4121 [47]	96 [32]	28 [23]	68 [37]	4025 [47]	375 [27]	3605 [51]
Age	Mean (SD)	36.7 (6.5)	37.1 (6.40)	37.1 (6.29)	37.2 (6.49)	36.7 (6.48)	36.7 (6.46)	36.7 (6.48)
Zygosity								
	Monozygotic	3129 [36]	95 [31]	36 [30]	59 [32]	3034 [34]	516 [38]	2518 [35]
	Dizygotic	2702 [31]	96 [32]	45 [37]	51 [28]	2606 [31]	390 [28]	2216 [31]
	Unknown zygosity	194 [2]	< 10 [1]	< 10 [2]	< 10 [1]	191 [2]	25 [2]	166 [2]
	Opposite sex	2770 [32]	110 [36]	38 [31]	72 [39]	2660 [31]	437 [32]	2223 [31]
Marital status								
	Married/cohabiting	6817 [78]	220 [72]	88 [73]	132 [72]	6597 [78]	1042 [76]	5555 [78]
	Single	1978 [22]	84 [28]	33 [27]	51 [28]	1894 [22]	326 [24]	1568 [22]
Children living at home								
	Yes	4994 [57]	163 [54]	61 [50]	102 [56]	4831 [57]	805 [59]	4026 [57]
	No	3801 [43]	141 [46]	60 [50]	81 [44]	3660 [43]	563 [41]	3097 [43]
Type of living area								
	City	3435 [39]	122 [40]	49 [40]	73 [40]	3313 [39]	533 [39]	2780 [39]
	Town and suburban	3708 [42]	115 [38]	49 [40]	66 [36]	3593 [42]	574 [42]	3019 [42]
	Rural	1652 [19]	67 [22]	23 [19]	44 [24]	1585 [19]	261 [19]	1324 [19]
Length of education								
	Shorter	4466 [51]	149 [49]	57 [47]	92 [50]	4317 [51]	704 [51]	3613 [51]
	Longer	4329 [49]	155 [51]	64 [53]	91 [50]	4174 [49]	664 [49]	3510 [49]
Work environment factors								
	High job demands	3397 [39]	200 [66]	79 [65]	121 [66]	3197 [38]	580 [42]	2617 [37]
	Low job demands	5397 [61]	104 [34]	42 [35]	62 [34]	5294 [62]	788 [58]	4506 [63]
	Low control	3791 [43]	149 [49]	59 [49]	90 [49]	3642 [43]	598 [44]	3044 [43]
	High control	5004 [57]	155 [51]	62 [51]	93 [51]	4894 [57]	770 [56]	4079 [57]
	Low support	3764 [43]	216 [71]	89 [74]	127 [69]	3548 [42]	610 [45]	2938 [41]
	High support	5031 [57]	88 [29]	32 [26]	56 [31]	4943 [58]	758 [55]	4185 [59]
	Job insecurity, (scale 1–4) mean (SD)	1.52 (0.75)	1.97 (0.92)	1.96 (0.95)	1.98 (0.91)	1.50 (0.74)	1.61 (0.80)	1.48 (0.73)
Symptoms of depression								
	CESD-scale, (scale 0–33) mean (SD)	5.8 (4.96)	10.1 (6.18)	11.5 (6.13)	9.17 (6.06)	5.6 (4.84)	6.98 (5.50)	5.39 (4.66)
Symptoms of burnout								
	Symptoms	1468 [17]	162 [53]	74 [61]	88 [48]	1306 [15]	392 [29]	914 [13]
	No symptoms	7327 (83)	142 [47]	47 [39]	95 [52]	7185 (85)	976 [71]	6209 (87)
Personality								
	Higher level of neuroticism	2216 [25]	157 [52]	70 [58]	87 [48]	2059 [24]	514 [38]	1545 [22]
	Lower level of neuroticism	6579 [75]	147 [48]	51 [42]	96 [52]	6432 [76]	854 [62]	5578 [78]

### Outcome

Sickness absence spells, 14 net days or longer, with diagnostic codes F30-F39 and F40-F48 according to the International statistical Classification of Diseases and Related Health Problems version-10 (ICD-10), were included and classified as sickness absence spells due to CMDs. A binary outcome variable was created with those who had at least one spell of sickness absence due to the above-mentioned diagnoses during the follow-up time (coded as 1) and those who had no sickness absence spell due to CMDs during the follow-up (coded as 0). The follow-up period ranged from survey response-date (once for each individual in 2005 or 2006) until the end of 2018.

### Exposures

Exposure to work-related violence or threats of violence, and work-related harassment or bullying, were measured using survey questions. First, the respondent answered if they during the last 12 months had problems due to work that made it hard to work or do daily chores at home, and if they responded yes to any of those two questions, the individual answered a subsequent question concerning how often these problems were caused by either violence/threats or harassment/bullying, respectively, on a 4-point response scale ranging from *often, sometimes, seldom, never to almost never.* For our analyses, dichotomous exposure variables were created by coding the first three response alternatives as 1 (*“yes”*) and the fourth as 0 (*“no”*). In addition, a combined dichotomous variable was created as an overall indicator of any *or* both types of exposure.

### Covariates

According to previous literature [42, 52, 53], several potential demographic, health-related and work-related confounders were identified and considered.

Sex (man or woman), age at baseline (continuous), marital status (married/cohabiting or single), having children living at home (yes or no), type of living area (city area, town/suburban area, or rural area) and level of education were included as covariates. For education, a binary variable was created by categorizing respondents into two groups with a lower education level (elementary school and/or vocational school, including residential college for adult education), or a higher education level (university, including military school and vocational university). This demographic information was extracted from LISA [51].

Health-related factors assessed in the STAGE survey included symptoms of depression and burnout. Depressive symptoms were measured using the Center for Epidemiological Studies–Depression (CES-D) scale [54]. The STAGE survey includes a validated short-form inventory with 11 questions [55] and the variable was handled as continuous. Symptoms of burnout was measured using a validated short form of Pines Burnout Measure [56], including three questions regarding symptoms of burnout, which has been shown to correlate strongly with the full Pines Burnout Measure [57]. Ratings were made along a 7-point scale ranging from 1 (never) to 7 (all the time), providing a total score ranging from 3 to 21. Following previous research, responses were dichotomized using a cut-off with scores above 4 corresponding to high symptoms of burnout [58].

Job demands, control and social support were measured using the Swedish Demand-Control-Support Questionnaire [59]. A binary variable for each dimension was created using a median cut-off. Additionally, job insecurity was included as a covariate and measured using three questions regarding fears of losing the job or being relocated within the organization. The mean value was calculated and the variable was handled as a continuous variable.

Finally, neuroticism was identified as a potential confounder or effect modifier. Neuroticism was measured using the short form of the Eysenck’s Personality questionnaire (EPQ), consisting of 9 items regarding behaviors and emotions during specific situations [60]. In the STAGE-survey, this short form of the questionnaire has been complemented with an additional 9 items from the full Eysenck Inventory [61], in order to provide a better (normal) distribution of the scale [62]. For each item, respondents were asked to indicate yes (1) or no (0) and then total sum scores were computed.

A binary variable was created for neuroticism, with the upper quartile being used as cut-off point when categorizing individuals into higher or lower level of neuroticism.

### Statistical analyses

The analyses of associations between exposure to work-related violence/threats, harassment/bullying, or exposure to either violence/threats or harassment/bullying (as a combined variable) and sickness absence due to CMDs were performed using logistic regression analyses. First, the crude Odds Ratios (OR) and associated 95% confidence intervals (CIs) of sickness absence due to CMDs were estimated, comparing odds in exposed with that of unexposed. As the overall study sample included twin pairs, the analyses were performed using cluster robust standard errors to account for non-independency.

Next, covariates were added sequentially to the analyses. In model 1 only sex and age were adjusted for, while in the fully adjusted model, model 5, the analyses were adjusted for all other identified covariates (see Table 2 for the different models). In addition, to assess the impact of personality, the fully adjusted model was also stratified by neuroticism and both multiplicative and additive interactions between the neuroticism variable and exposures were tested. These multiplicative interactions were tested by including an interaction term in the models, while additive interactions were measured by the relative excess risk due to interaction (RERI), attributable proportion (AP) and synergy index (S), which were calculated in accordance with previous literature [63].

**Table 2:** Logistic regression analyses of exposure to work-related offensive behaviors and SA due to CMDs^¤^. *n* = 8795

Exposure	Crude model OR (95% CI)	Model 1^a^ OR (95% CI)	Model 2^b^ OR (95% CI)	Model 3^c^ OR (95% CI)	Model 4^d^ OR (95% CI)	Model 5^e^ OR (95% CI)
Violence/threats	3.44 (2.55–4.64) *	3.18 (2.33–4.35) *	3.15 (2.31–4.32) *	2.26 (1.63–3.14) *	2.11 (1.52–2.95) *	2.15 (1.54–2.99) *
Harassment/bullying	2.92 (2.17–3.95) *	2.59 (1.90–3.53) *	2.56 (1.88–3.49) *	1.67 (1.21–2.31) *	1.52 (1.10–2.11) *	1.51 (1.09–2.09) *
Violence/threats *and/or* harassment/bullying	3.44 (2.71–4.37) *	3.11 (2.43–3.98) *	3.08 (2.41–3.95) *	2.11 (1.63–2.75) *	1.98 (1.52–2.59) *	1.98 (1.52–2.59) *

^¤^ICD10 diagnoses F30-F39 F40-F48. OR: Odds ratio. CMD: Common mental disorder. SA: Sickness absence

*p < 0.05

^a^ Model 1 adjusted for sex, age

^b^ Model 2 adjusted for sex, age, marital status, children, level of education, type of living area

^c^ Model 3 adjusted for sex, age, marital status, children, level of education, type of living area, self-reported symptoms of depression and burnout

^d^ Model 4 adjusted for sex, age, marital status, children, level of education, type of living area, self-reported symptoms of depression and burnout, work environment factors (demands, control, support, job insecurity)

^e^ Model 5 adjusted for sex, age, marital status, children, level of education, type of living area, self-reported symptoms of depression and burnout, work environment factors (demands, control, support, job insecurity) and neuroticism (dichotomous variable)

To assess the impact of any familial factors on the association between work-related violence/threats or harassment/bullying and sickness absence due to CMDs, co-twin control analyses using conditional logistic regression were performed [48]. Only complete pairs of MZ and same-sex DZ twins who were discordant (i.e., different) in terms of the exposure of interest were included. The sample used for analysis of exposure to violence and/or threats included 41 complete MZ and DZ twin pairs, whereas the sample used for analysis of exposure to harassment/bullying included 50 complete twin pairs. Furthermore, the sample used for analysis of exposure to any of these types of exposure included 76 complete twin pairs, discordant on exposure to violence/threats *or* harassment/bullying.

The co-twin control analyses allowed us to control for age, sex, and familial factors, that is, genetic and shared environmental factors. To investigate whether genetics or shared environment was the strongest influence, the analyses were further stratified on zygosity. However, due to low number of complete exposure discordant twin pairs in the present study, only results from analyses where both MZ and DZ twins were included are presented.

The statistical analyses were performed using STATA version 17.

## Results

### Descriptive statistics

The proportion of women in the study sample was slightly higher than that of men (53% and 47% respectively), and the mean age was 37 years. Most participants lived in either cities (39%) or towns and suburbs (42%) and about half reported having a university education (49%). Table 1 and S1-2 show descriptive statistics, for the whole sample, stratified on exposure to violence and/or threats *and*/*or* harassment (Table 1), stratified on exposure to violence and/or threats (Table S1), and stratified on exposure to harassment (Table S2). Tables S1-2 can be found in the supplement.

### Logistic regression analyses on the whole sample

In the logistic regression analyses adjusting for sex, age, family situation, level of education and type of living area, the OR for sickness absence due to CMDs following exposure to work-related violence and/or threats of violence was 3.15 (95% CI 2.31–4.32). Similar results were found for exposure to work-related harassment (OR 2.56, 95% CI 1.88–3.49) and the combined exposure variable (OR 3.08, 95% CI 2.41–3.95) in the adjusted model. When the model was further adjusted for symptoms of depression, symptoms of burnout and psychosocial work environment factors (e.g., job demands, control, social support and job insecurity), the ORs of sickness absence due to CMDs were reduced for all types of exposure (violence/threats: OR 2.11, 95% CI 1.52–2.95, harassment: OR 1.52, 95% CI 1.10–2.11 and the combined variable: OR 1.98, 95% CI 1.52–2.59), but the CIs were narrower and the results still statistically significant (Table 2).

### Co-twin control analyses on discordant twins

The OR for the association between work-related violence/threats and sickness absence due to CMDs was attenuated and not statistically significant in the co-twin control analysis (OR 2.0, CI 0.79–5.07), when contrasted with the analysis of the whole study sample adjusted for sex and age (OR 3.18, 95% CI 2.33–4.35). A similar pattern was found for the associations between work-related harassment and sickness absence due to CMDs in the co-twin control analysis (OR 1.56, CI 0.66–3.66 compared to OR 2.59, 95% CI 1.90–3.53 in the analysis of the whole study sample), and also for the combined variable of violence/threats *and/or* harassment/bullying with sickness absence due to CMDs (Table 3).

**Table 3:** Conditional logistic regression analyses of twin samples discordant for exposure to work-related offensive behaviors

Exposure	Logistic regression analyses of full study sample		Conditional logistic regression analyses among MZ and same-sex DZ twins discordant for exposure
Violence/threats	*n*	*n*	*n, complete pairs*
	8795	8795	41
	Crude model OR (95% CI)	Model 1^a^ OR (95% CI)	Co-twin control OR (95% CI)
	3.44 (2.55–4.64) *	3.18 (2.33–4.35) *	2.0 (0.79–5.07)
Harassment/bullying	*n*	*n*	*n, complete pairs*
	8795	8795	50
	Crude model OR (95% CI)	Model 1^a^ OR (95% CI)	Co-twin control OR (95% CI)
	2.92 (2.17–3.95) *	2.59 (1.90–3.53) *	1.56 (0.66–3.66)
Violence/threats *and/or* harassment/bullying	*n*	*n*	*n, complete pairs*
	8795	8795	76
	Crude model OR (95% CI)	Model 1^a^ OR (95% CI)	Co-twin control OR (95% CI)
	3.44 (2.71–4.37) *	3.11 (2.43–3.98) *	1.85 (0.93–3.66)

*p < 0.05. OR: Odds ratio

^a^ Model 1 adjusted for sex, age

To assess separately the impact of genetics and shared early environment, the co-twin control analyses were also stratified on zygosity. The results showed a tendency of a lower OR for the MZ twin pairs compared to the DZ twin pairs for the associations between all three types of exposure and sickness absence due to CMDs, but precision was low due to limited number of complete pairs when stratified by zygosity (data not shown).

### Interaction analyses

To investigate the impact neuroticism may have on the association of exposure to violence/threats and/or harassment/bullying with sickness absence due to CMDs, the fully adjusted regression models were adjusted for neuroticism (Table 3, model 5) and the results remained almost unchanged.

The fully adjusted models were further stratified by neuroticism. Among individuals with lower levels of neuroticism (i.e., those scoring lower than the upper quartile), the odds of sickness absence due to CMDs following exposure to violence/threats was particularly high (OR 2.48, 95% CI 1.57–3.94), compared with their unexposed references. Among individuals with higher levels of neuroticism (i.e., those scoring within the upper quartile), the odds of sickness absence due to CMDs following exposure to violence/threats was also elevated (OR 1.85, 95% CI 1.16–2.94), compared with their unexposed references. There was a tendency towards the same pattern of results when looking at exposure to harassment/bullying (data not shown). When investigating the interaction between level of neuroticism and exposure to work-related violence/threats and/or harassment, by adding an interaction term in the regression models, results were not statistically significant, indicating no multiplicative interaction between neuroticism and exposure to offensive behaviors at work (results not shown). However, the analysis of the relative excess risk due to interaction (RERI) between neuroticism and exposure to work-related violence or threats resulted in a RERI of < 0, *p* < 0.05, indicating a higher risk of SA due to CMD in the group scoring lower on neuroticism (individuals scoring lower than the upper quartile on the scale). The additive interaction was further calculated through the measure of attributable proportion (AP) and synergy index (S), and the results of the three different measures of additive interaction were consistent and indicated a negative interaction on the additive scale (AP < 0 and S < 1). The same pattern was found when investigating the additive interaction effect between neuroticism and work-related harassment, however the RERI estimate was not statistically significant (see Table 4 for the results of the additive interaction analyses).

**Table 4:** Measures of interaction on additive scale between the exposure variables and neuroticism

	Exposure to violence/threats	Exposure to harassment/bullying	Exposure to violence/threats and/or harassment/bullying
RERI	-0.75*	-0.33	-0.64
AP^¤^	-0.35	-0.22	-0.32
S^¤^	0.80	0.01	0.75

*p < 0.05

^¤^Confidence intervals and *p*-values not available

## Discussion

The results of this population-based, prospective twin study point to an association between exposure to work-related offensive behaviors and sickness absence due to CMDs. However, our findings suggest that this association may partially be influenced by familial factors (i.e., genetics and shared early environment), which to the best of our knowledge has not been reported before. Furthermore, previous history of depression, burnout and work-related psychosocial environment seems to influence the associations between work-related offensive behaviors and sickness absence due to CMDs. In addition, the risk of sickness absence due to CMDs appear to be particularly elevated among individuals with low levels of neuroticism.

Overall, our results showing an association between exposure to offensive behaviors at work and sickness absence due to CMDs is consistent with results of a previous study on workplace violence [25]. However, the results from the co-twin control analyses suggest that the association between exposure to offensive behaviors at work and sickness absence due to CMDs may be at least partially confounded by familial factors. This finding is in line with previous studies addressing the role of familial factors in the associations between factors in the psychosocial work environment and mental health outcomes. Blom and colleagues (2013) found that familial factors influenced the association between social support and symptoms of burnout [41]. Another study by Mather et al. (2015), indicated a similar confounding by familial factors in the association between social support and sickness absence due to mental disorders [42]. Taken together, the results from previous studies indicate that associations between exposure to social stressors and mental health outcomes, including sickness absence due to mental disorders, may be confounded by familial factors. Extrapolating from this, it is plausible that familial factors play a similar role in the association between work-related offensive behaviors and sickness absence due to common mental disorders, which makes this study a contribution to the existing knowledge on the role of familial factors, not often accounted for in observational studies.

In twin studies, unmeasured familial factors refer to both genetics and early environmental factors shared by the twins. Previous studies have indicated that certain early environmental factors as well as genetics seem to have an impact on individuals’ disposition and vulnerability to disease. For instance, the experience of multiple childhood adversities, such as household substance abuse, criminality, economic adversity or parental mental illness, have been found to increase the risk for negative health outcomes, including mental ill-health [64]. Genetics also seem to influence mental disorders such as major depression, and a previous study investigating the relative importance of genetic and environmental factors for sickness absence have found that genetic factors account for approximately 30% of the total variance in sickness absence due to any diagnosis [65, 66]. Furthermore, early environmental factors such as childhood adversity have been linked to offensive behaviors at work [39, 40], and genetic factors that have been found to influence work characteristics such as job demands/control [67, 68] may also be linked to offensive behaviors at work [26, 27]. These results suggest that both genetic factors and shared environmental factors can be common causes of the dependent and independent variables included in the present study and thereby confound the association of interest. To increase the understanding of whether genetics or shared environment have the strongest impact on the association of interest, the analyses were additionally stratified on zygosity. The results showed a tendency of lower odds among the group of MZ twin pairs compared to DZ twin pairs, suggesting mainly genetic influences on the association. However, when analyses were stratified on zygosity the groups of MZ and DZ twins were small resulting in low precision.

The precision was also relatively low in the overall co-twin analyses. Consequently, a causal association between exposure and outcome can neither be confirmed or ruled out in this study, nor can reverse causality be ruled out. To further assess the potential causality and the importance of familial factors (genetics or shared early environment) on the associations, more studies including larger twin samples are needed.

Neuroticism did not markedly reduce ORs when added as a covariate in the models. Instead, when the models were stratified on neuroticism, we found that the association of interest were more marked among individuals with a lower level of neuroticism. This suggest that individuals with lower levels of neuroticism have higher odds of sickness absence due to CMDs when exposed to offensive behaviors at work, even though people with high level of neuroticism are expected to have both higher exposure to adversities [35, 36] and a higher tendency to react with strong negative emotions when exposed to adversities such as negative events in the work environment. But, our result is in line with a study by Booth et al. (2013), showing that individuals who score lower in neuroticism have a higher risk of depression when exposed to low control at work [69]. High levels of neuroticism are usually considered a risk factor for negative health outcomes, however, in some situations, neuroticism may be adaptive. The concept of “healthy neuroticism”, emphasize the functional benefits of vigilance and worry in threatening situations [70]. Studies have suggested that high levels of neuroticism may actually boost stress resilience [71] and promote recovery after highly stressful periods [72]. Although neuroticism seems related to less problem-solving and to some inappropriate problem-solving strategies, neuroticism has also been related to more support-seeking [73]. Support has, in turn, been suggested to reduce the impact of e.g. workplace bullying on sickness absence [74]. It is also possible that neuroticism is associated with e.g. more active, direct or confrontative coping patterns indicated as more effective in reducing the impact of work stressors [75]. These provide potential explanations as to why the results of the present study show a more marked association between exposure to adverse behaviors at work and sickness absence among individuals scoring low on neuroticism.

Other studies investigating the influence of neuroticism on the effect of exposure to workplace bullying on employee well-being have on the other hand shown contradicting results [76]. Hence, further research is needed in order to clarify the role of personality in the associations investigated in the present study.

Furthermore, previous studies have shown that exposure to bullying or harassments at work, may involve changes in individual dispositions including reduced extraversion and openness and increased neuroticism [36, 77, 78]. This suggests that personality may also be an intermediate variable/mediator in the association. However, whether neuroticism is in fact an effect modifier/moderator or can act as a mediator in the association between exposure to violence/threats or harassment/bullying and sickness absence due to CMDs cannot be determined in our study since the exposures and neuroticism were measured simultaneously at baseline.

### Strengths and limitations

The present study includes both strengths and limitations. One of the most distinctive strengths involve the use of a twin sample. The fact that the sample consists of MZ and DZ twin pairs give a possibility to assess whether familial factors play a role in the association between exposure to work-related offensive behaviors and sickness absence due to CMDs. This makes the study unique. The application of a co-twin control design in attempts to investigate causal effects is a strong alternative design when the use of randomized controlled trials is unfeasible or unethical. However, in the present study, the number of twin pairs discordant on the exposures were small, making the estimates uncertain. Due to lack of power in the co-twin control analyses, the results are merely tentative.

The individual-level regression analyses, performed on all twins treated as unrelated individuals, were performed on a relatively large sample of Swedish twins, which can be considered a strength. Mostly, twins are no different from singletons and previous studies using data derived from the Swedish Twin Registry have found that the twin sample was generalizable in studies on CMDs [79] as well as in studies investigating disability pension due to mental disorders [80].

The fact that the present study used self-reported data as measure of exposure may impose a risk for measurement error and errors due to social desirability. However, the risk of common method bias is reduced due to the fact that a combination of self-report data and register data derived from good-quality national registries is used and thus, the outcome is independent from the exposure measurement. This, in combination with the prospective study design can be considered a major strength.

The sample included a large proportion of highly educated individuals. Although our models were adjusted for education we cannot exclude the possibility of selection bias, which may restrict generalizability.

A concern regarding the use of twin studies as well as other family-based designs, is the fact that such designs, despite the obvious advantages may impose a risk for bias from non-shared confounders and measurement errors [81, 82]. This risk can primarily be explained by the fact that when selecting twin pairs discordant in the exposure of interest, the selection itself may result in twins who show within-pair differences also in terms of non-shared confounders, alongside differences caused by measurement error [81].

In Sweden, the sickness insurance covers all who live in the country, who are above 16 years of age and have at least a minimum annual income from work and the minimum criteria to be eligible to sickness absence benefits is fulfilled if someone has a disease or injury in such a way that the ability to work is reduced by at least 25% [14]. All spells of sickness absences lasting longer than 14 days are included in the register, eliminating the risk of recall bias and loss to follow-up. However, there are a few limitations. Firstly, we only had access to the first diagnosis; if a diagnosis change there might be cases of sickness absence due to CMDs that are not included. Another limitation is that shorter sickness absence spells (< 14 days) are not included.

In the present study, several possible confounding variables were included in the regression models performed on the whole sample of twins treated as singletons. This can be considered a strength, and the possibility to also include potential confounders that are seldom accounted for such as familial factors in the conditional regression analyses is a major strength. Nevertheless, the risk of bias due to unmeasured and residual confounding cannot be completely ruled out. When symptoms of depression and burnout at baseline were added as covariates in the analyses performed on the whole sample, where twins were treated as singletons, the estimates were reduced. This indicates that symptoms of depression and burnout may partly explain/confound the association. However, these variables may potentially mediate the association. If so, adjusting for these factors may yield biased estimates. To increase the probability that the exposure and covariates temporally preceded the outcome, we included individuals with symptoms of depression or burnout assessed at baseline, while those on sickness absence due to common mental disorders at the time of survey response were excluded.

A limitation related to the role of neuroticism in the studied associations is that it is not possible to determine whether the score of neuroticism were the same before and after exposure, since personality is measured at a single timepoint. For instance, neuroticism scores can be influenced by the exposure investigated [77], which makes it difficult to fully determine the role of neuroticism in the association between exposure and outcome.

### Concluding remarks

In this prospective twin study, we found that the association between exposures to work-related offensive behaviors and sickness absence due to CMDs may at least partially be confounded by genetics and shared early environment, and furthermore that there might be an interaction between exposures and neuroticism in relation to sickness absence due to CMDs. Thus, in future studies investigating the associations of the psychosocial work environment and sickness absence, familial factors, as well as neuroticism, should be considered.

### Electronic supplementary material

Below is the link to the electronic supplementary material.

Supplementary Material 1

## Data Availability

The data that support the findings of this study are available from the original sources: the Swedish Twin Registry, Statistics Sweden, the Swedish Social Insurance Agency and the Swedish National Board of Health and Welfare. Restrictions apply to the availability of the data used in this study, based on the Swedish Twin project Of Disability pension and Sickness absence (STODS). The data, which were used with ethics approval for the current study is not publicly available.

## Declarations

### Disclaimer

The funders were not involved in the study design, collection, or analysis of the data, interpretation of the results, writing of the paper nor in decisions about the manuscript submission.

## Abbreviations

AP: Attributable Proportion
CES-D: Center for Epidemiological Studies - Depression
CI: Confidence Interval
CMD: Common Mental Disorder
DNA: Deoxyribonucleic Acid
DZ: Dizygotic
EPQ: Eysenck’s Personality Questionnaire
ICD: International Classification of Disease
LISA: The Longitudinal Integrated database for Health Insurance and Labor Market Studies
MiDAS: Micro Data for Analysis of Social Insurance
MZ: Monozygotic
OR: Odds Ratio
RERI: Relative Excess Risk due to Interaction
S: Synergy index
SA: Sickness Absence
STAGE: The Study of Swedish Twin Adults: Genes and Environment
STODS: The Swedish Twin Project of Disability Pension and Sickness Absence
