# Supplementary material for: The role of familial factors and neuroticism in the association between exposure to offensive behaviors at work and long-term sickness absence due to common mental disorders - a prospective twin study
Source: BMC Public Health. 2024 Jun 1;24:1473. doi: 10.1186/s12889-024-19000-z (PMC11143713; doi:10.1186/s12889-024-19000-z)
Supplement: Supplementary file 1 — Supplementary Material 1 [file 12889_2024_19000_MOESM1_ESM.docx]

***Supplementary materials***

Table S1. Descriptive statistics including the full study sample, stratified on outcome and exposure to work-related violence/threats

|  |  | **Total** | ***Exposure to work-related violence and/or threats*** | | | ***No exposure to work-related violence and/or threats*** | | |
| --- | --- | --- | --- | --- | --- | --- | --- | --- |
| **Background factors** |  |  | ***All***  ***n (%)*** | ***Sickness absence due to CMD***  ***n (%)*** | ***No Sickness absence due to CMD***  ***n (%)*** | ***All***  ***n (%)*** | ***Sickness absence due to CMD***  ***n (%)*** | ***No Sickness absence due to CMD***  ***n (%)*** |
|  |  | **8795** | 186 | 75 | 111 | 8609 | 1414 | 7195 |
| **Sex** |  |  |  |  |  |  |  |  |
|  | Women | 4674 (53) | 123  (66) | 56  (75) | 67  (60) | 4551  (53) | 1030  (73) | 3521  (49) |
|  | Men | 4121 (47) | 63  (34) | 19  (25) | 44  (40) | 4058  (47) | 384  (27) | 3674  (51) |
|  |  |  |  |  |  |  |  |  |
| **Age** | Mean (SD) | 36,7 (6.5) | 37.4  6.32) | 37.4 (5.73) | 37.3 (6.7) | 36.7 (6.48) | 36.7 (6.48) | 36.7 (6.48) |
|  |  |  |  |  |  |  |  |  |
| **Zygosity** |  |  |  |  |  |  |  |  |
|  | Monozygotic | 3129 (36) | 53  (28) | 20  (27) | 33  (30) | 3076  (36) | 532  (38) | 2544  (36) |
|  | Dizygotic | 2702 (31) | 57  (31) | 30  (40) | 27  (24) | 2645  (31) | 405  (28) | 2240  (31) |
|  | Unknown zygosity | 194 (2) | <10  (2) | <10  (3) | <10  (1) | 191  (2) | 25  (2) | 166  (2) |
|  | Opposite sex | 2770 (32) | 73  (39) | 23  (31) | 50  (45) | 2697  (31) | 452  (32) | 2245  (31) |
|  |  |  |  |  |  |  |  |  |
| **Marital status** |  |  |  |  |  |  |  |  |
|  | Married/cohabiting | 6817  (78) | 135  (73) | 58  (77) | 77  (69) | 6682  (78) | 1072  (76) | 5610  (78) |
|  | Single | 1978  (22) | 51  (27) | 17  (23) | 34  (31) | 1927  (22) | 342  (24) | 1585  (22) |
|  |  |  |  |  |  |  |  |  |
| **Children living at home** |  |  |  |  |  |  |  |  |
|  | Yes | 4994 (57) | 103  (56) | 40  (53) | 63  (57) | 4891 (57) | 826  (58) | 4065  (56) |
|  | No | 3801 (43) | 83  (44) | 35  (47) | 48  (43) | 3718  (43) | 589  (42) | 3130  (44) |
|  |  |  |  |  |  |  |  |  |
| **Type of living area** |  |  |  |  |  |  |  |  |
|  | City | 3435 (39) | 73  (39) | 30  (40) | 43  (39) | 3362 (39) | 552  (39) | 2810  (39) |
|  | Town and suburban | 3708 (42) | 71  (38) | 30  (40) | 41  (37) | 3637 (42) | 593  (42) | 3044  (42) |
|  | Rural | 1652 (19) | 42  (23) | 15  (20) | 27  (24) | 1610  (19) | 269  (19) | 1341  (19) |
|  |  |  |  |  |  |  |  |  |
| **Length of education** |  |  |  |  |  |  |  |  |
|  | Shorter | 4466  (51) | 93  (50) | 35  (47) | 58  (52) | 4373  (51) | 726  (51) | 3647  (51) |
|  | Longer | 4329  (49) | 93 (50) | 40  (53) | 53  (48) | 4236  (49) | 688  (49) | 3548  (49) |
|  |  |  |  |  |  |  |  |  |
| **Work environment factors** |  |  |  |  |  |  |  |  |
|  | High job demands | 3397 (39) | 121  (65) | 50  (67) | 71  (64) | 3276  (38) | 609  (43) | 2667  (37) |
|  | Low job demands | 5397  (61) | 65  (35) | 25  (33) | 40  (36) | 5333  (62) | 805  (57) | 4528  (63) |
|  | Low control | 3791 (43) | 90  (48) | 34  (45) | 56  (50) | 3701  (43) | 623  (44) | 3078  (43) |
|  | High control | 5004  (57) | 96  (52) | 41  (55) | 55  (50) | 4908  (57) | 791  (56) | 4117  (57) |
|  | Low support | 3764 (43) | 119  (64) | 48  (64) | 71  (64) | 3645  (42) | 651  (46) | 2994  (42) |
|  | High support | 5031  (57) | 67  (36) | 27  (36) | 40  (36) | 4964  (58) | 763  (54) | 4201  (58) |
|  | Job insecurity, (scale 1-4) mean (SD) | 1.52 (0.75) | 1.98  (0.96) | 1.97 (1.01) | 1.99 (0.92) | 1.51  (0.74) | 1.62 (0.80) | 1.49 (0.73) |
|  |  |  |  |  |  |  |  |  |
| **Symptoms of depression** |  |  |  |  |  |  |  |  |
|  | CESD-scale, (scale 0-33) mean (SD) | 5.8 (4.96) | 9.5  (6.10) | 10.5 (5.97) | 8.8 (6.09) | 5.7  (4.90) | 7.18  (5.62) | 5.4 (4.69) |
|  |  |  |  |  |  |  |  |  |
| **Symptoms of burnout** |  |  |  |  |  |  |  |  |
|  | Symptoms | 1468 (17) | 92  (49) | 43  (57) | 49  (44) | 1376  (16) | 423  (30) | 953  (13) |
|  | No symptoms | 7327  (84) | 94  (51) | 32  (43) | 62  (56) | 7233  (84) | 991  (70) | 6242  (87) |
|  |  |  |  |  |  |  |  |  |
| **Personality** |  |  |  |  |  |  |  |  |
|  | Higher level of neuroticism | 2216 (25) | 82  (44) | 37  (49) | 45  (41) | 2134  (25) | 547  (39) | 1587  (22) |
|  | Lower level of neuroticism | 6579  (75) | 104  (56) | 38  (51) | 66  (59) | 6475  (75) | 867  (61) | 5608  (78) |
|  |  |  |  |  |  |  |  |  |

Table S2. Descriptive statistics including the full study sample, stratified on outcome and exposure to work-related harassment/bullying

|  |  | **Total** | ***Exposure to work-related harassment/bullying*** | | | ***No exposure to work-related harassment/bullying*** | | |
| --- | --- | --- | --- | --- | --- | --- | --- | --- |
| **Background factors** |  |  | ***All***  ***n (%)*** | ***Sickness absence due to CMD***  ***n (%)*** | ***No Sickness absence due to CMD***  ***n (%)*** | ***All***  ***n (%)*** | ***Sickness absence due to CMD***  ***n (%)*** | ***No Sickness absence due to CMD***  ***n (%)*** |
|  |  | **8795** | 194 | 71 | 123 | 8601 | 1418 | 7183 |
| **Sex** |  |  |  |  |  |  |  |  |
|  | Women | 4674 (53) | 135  (70) | 56  (79) | 79  (64) | 4539  (53) | 1030  (73) | 3509  (49) |
|  | Men | 4121 (47) | 59  (30) | 15  (21) | 44  (36) | 4062  (47) | 388  (27) | 3674  (51) |
|  |  |  |  |  |  |  |  |  |
| **Age** | *Mean (SD)* | 36,7 (6.5) | 37.3 (6.38) | 36.9  (6.64) | 37.5  (6.25) | 36.7  (6.48) | 36.7  (6.44) | 36.7  (6.49) |
|  |  |  |  |  |  |  |  |  |
| **Zygosity** |  |  |  |  |  |  |  |  |
|  | Monozygotic | 3129 (36) | 64  (33) | 22  (31) | 42  (34) | 3065  (36) | 530  (37) | 2535  (35) |
|  | Dizygotic | 2702 (31) | 58  (30) | 24  (34) | 34  (28) | 2644  (31) | 411  (29) | 2333  (32) |
|  | Unknown zygosity | 194 (2) | <10 (0,5) | <10  (1) | <10  (0) | 193  (2) | 26  (2) | 167  (2) |
|  | Opposite sex | 2770 (32) | 71  (37) | 24  (34) | 47  (38) | 2699  (31) | 451  (32) | 2248  (31) |
|  |  |  |  |  |  |  |  |  |
| **Marital status** |  |  |  |  |  |  |  |  |
|  | Married/cohabiting | 6817  (78) | 138  (71) | 49  (69) | 89  (72) | 6679  (78) | 1081  (76) | 5598  (78) |
|  | Single | 1978  (22) | 56  (29) | 22  (31) | 34  (28) | 1922  (22) | 337  (24) | 1585  (22) |
|  |  |  |  |  |  |  |  |  |
| **Children living at home** |  |  |  |  |  |  |  |  |
|  | Yes | 4994 (57) | 106 (55) | 37  (52) | 69  (56) | 4888  (57) | 829  (58) | 4059  (57) |
|  | No | 3801 (43) | 88 (45) | 34  (48) | 54  (44) | 3713  (43) | 589  (42) | 3124  (43) |
| **Type of living area** |  |  |  |  |  |  |  |  |
|  | City | 3435 (39) | 80  (41) | 29  (41) | 51  (41) | 3355  (39) | 553  (39) | 2802  (39) |
|  | Town and suburban | 3708 (42) | 72  (37) | 29  (41) | 43  (35) | 3636  (42) | 594  (42) | 3042  (42) |
|  | Rural | 1652 (19) | 42  (22) | 13  (18) | 29  (23) | 1610  (19) | 271  (19) | 1339  (19) |
|  |  |  |  |  |  |  |  |  |
| **Length of education** |  |  |  |  |  |  |  |  |
|  | Shorter | 4466  (51) | 96  (49) | 36  (51) | 60  (49) | 4370  (51) | 725  (51) | 3645  (51) |
|  | Longer | 4329  (49) | 98  (51) | 35  (49) | 63  (51) | 4231  (49) | 693  (49) | 3538  (49) |
|  |  |  |  |  |  |  |  |  |
| **Work environment factors** |  |  |  |  |  |  |  |  |
|  | High job demands | 3397 (39) | 137  (71) | 50  (70) | 87  (70) | 3260  (38) | 609  (43) | 2651  (37) |
|  | Low job demands | 5397  (61) | 57  (29) | 21  (30) | 36  (30) | 5341  (62) | 809  (57) | 4532  (63) |
|  | Low control | 3791 (43) | 101  (52) | 38  (53) | 63  (51) | 3690  (43) | 619  (44) | 3071  (43) |
|  | High control | 5004  (57) | 93  (48) | 33  (47) | 60  (49) | 4911  (57) | 799  (56) | 4112  (57) |
|  | Low support | 3764 (43) | 161  (83) | 64  (90) | 97  (79) | 3603  (42) | 635  (45) | 2968  (41) |
|  | High support | 5031  (57) | 33  (17) | 7  (10) | 26  (21) | 4998  (58) | 783  (55) | 4215  (59) |
|  | Job insecurity, (scale 1-4) mean (SD) | 1.52 (0.75) | 2.08 (0.90) | 2.03  (0.92) | 2.12 (0.90) | 1.50  (0.74) | 1.62 (0.81) | 1.49 (0.73) |
|  |  |  |  |  |  |  |  |  |
| **Symptoms of depression** |  |  |  |  |  |  |  |  |
|  | CESD-scale, (scale 0-33) mean (SD) | 5.8 (4.96) | 10.6  (6.35) | 12.8  (6.08) | 9.38  (6.19) | 5.7  (4.86) | 7.0 (5.53) | 5.4 (4.68) |
|  |  |  |  |  |  |  |  |  |
| **Symptoms of burnout** |  |  |  |  |  |  |  |  |
|  | Symptoms | 1468 (17) | 108  (56) | 47  (66) | 61  (50) | 1360  (16) | 419  (30) | 941  (13) |
|  | No symptoms | 7327  (83) | 86  (44) | 24  (34) | 62  (50) | 7241  (84) | 999  (70) | 6242  (87) |
|  |  |  |  |  |  |  |  |  |
| **Personality** |  |  |  |  |  |  |  |  |
|  | Higher level of neuroticism | 2216 (25) | 112  (56) | 47  (66) | 65  (53) | 2104  (24) | 537  (38) | 1567  (22) |
|  | Lower level of neuroticism | 6579  (75) | 82  (42) | 24  (34) | 58  (47) | 6497  (76) | 881  (62) | 5616  (78) |
|  |  |  |  |  |  |  |  |  |
